# Ethnomedicine and dominant medicine in multicultural Australia: a critical realist reflection on the case of Korean-Australian immigrants in Sydney

**DOI:** 10.1186/1746-4269-3-1

**Published:** 2007-01-03

**Authors:** Gil-Soo Han, Harry Ballis

**Affiliations:** 1 School of Arts and Sciences, Monash University Malaysia, No. 2 Jalan Universiti, Bandar Sunway, 46150 Petaling Jaya, Selangor, Malaysia; 2 Office of Pro-Vice-Chancellor, Monash University Gippsland Campus, Churchill, Victoria 3842, Australia

## Abstract

**Background:**

Viewed through the micro focus of an interpretive lens, medical anthropology remains mystified because interpretivist explanations seriously downplay the given context in which individual health seeking-behaviours occur. This paper draws upon both the interpretivist and political economy perspectives to reflect on the ethno medical practices within the Korean-Australian community in Sydney.

**Methods:**

We draw on research data collected between 1995 and 1997 for an earlier study of the use of biomedical and traditional medicine by Korean-Australians in Sydney. A total of 120 interviews were conducted with a range of participants, including biomedical doctors, traditional health professionals, Korean community leaders and Korean migrants representing a range of socio-economic backgrounds and migration patterns.

**Results and Discussion:**

First, the paper highlights the extent to which the social location of migrants in a host society alters or restructures their initial cultural practices they bring with them. Second, taking *hanbang *medicine in the Korean-Australian community as an illustrative case, the paper explores the transformation of the dominant biomedicine in Australia as a result of the influx of ethnomedicine in the era of global capitalism and global movement.

**Conclusion:**

In seeking to explain the popularity and supply of alternative health care, it is important to go beyond the culture of each kind of health care itself and to take into consideration the changes occurring at societal, national and global levels as well as consequential individual response to the changes. New social conditions influence the choice of health care methods, including herbal/alternative medicine, health foods and what are often called New Age therapies.

## Background

There is no simple answer to the question, what happens when oriental medicine is transplanted and practised in an Australian context? The question touches on a number of issues relating to the application of traditional non-Western medical practices in western societies [1,2]. Ethnomedicine in its given oriental cultural and historical contexts does not cease to exert itself upon transplantation into its new social context, but continues to influence health care choices and practices. The life experiences of the immigrants in a foreign land in general, and their working lives in particular, significantly influence their attitudes to health care and the range of health care they seek. How the host society and dominant biomedical practitioners view ethnomedicine, and who can satisfactorily provide the particular migrant community with ethnomedicine from the viewpoints of both providers and consumers also affect migrants' attitudes towards traditional medicine and health care. The resurgence of traditional medicine in contemporary western societies is closely linked to the key social scientific question of how to understand the juxtaposition of tradition and modernity at a particular historical juncture.

The most prominent attempts to explain the role of ethnomedicine in contemporary societies bear the influence of the interpretivist strands of social theories eg., [3,4]. Interpretivist explanations essentially focus on agency and individual choice without necessarily considering the given social and historical context. Morsy [5] and Singer [6] lamented the 'missing link in medical anthropology' and called for a critical medical anthropology. They saw critical medical anthropology as providing a distinct framework for understanding patterns of individual health care use in the social, political and economic contexts of society.

The strands of social theory in the interpretivist tradition and now broadly influenced by postmodernism and poststructuralism, have made a significant contribution to medical social science, by alerting researchers to the voices of individual agents. However, in the same way that predominantly structural perspectives do not fully capture the social reality as it is experienced by individual actors, the range of interpretivist theories provide only a partial perspective of the same reality. Viewed through the micro focus of an interpretive lens, medical anthropology remains mystified because interpretivist explanations seriously downplay the given context in which individual health-seeking behaviours occur.

Despite Singer's [7] renewed argument for the usefulness of medical anthropology and consequent suggestion of how social science can contribute to people's health needs, the age of critical medical anthropology is yet to be realised [8]. Critics of the medical anthropology of political economy argue that focusing on socio-political concerns depersonalizes and prevents people's voices from being heard e.g., [9]. Consequently some researchers, including Bhaskar [10] and Collier [11], opt for a combination of interpretivism and political economy to overcome such limitations. However, while researchers are not always able to consider all factors pertaining to a phenomenon being studied [12,13], it is also true that some theories are better able to explain social phenomena than others. Giddens' [14] structuration theory and Bhaskar's [10] critical realism are two theoretical frameworks that highlight the importance of agency while simultaneously providing close description of the social phenomena under consideration. This paper uses Bhaskar's critical realism, which acknowledges the pre-existence of structure prior to any actions taken by agency and draws upon both the interpretivist and political economy perspectives to reflect on the ethno-medical practices in the Korean-Australian community in Sydney [8].

A recent study of traditional medicines in Mexico notes that although there is competition among non-biomedical practitioners, a spirit of solidarity exists among a majority of practitioners promoting traditional health both with their patients and with fellow practitioners [[15]: 19]. The reason for such solidarity and how it might change in the future would be an important topic to observe. At this point in time the promotion of traditional medicine in Mexico is still in its infancy and not fully geared to global medical capitalism as it is in Korea. The socio-economic conditions in Mexico will most likely change in the next couple of decades and follow a similar pattern of development as has been evident in Korea. We can anticipate, therefore, that ethnomedicine in Mexico will achieve a level of market sophistication along similar lines to what occurred with *hanbang *medicine in Korea. The alternative medicine sector in Mexico already appears to be growing in appeal among the medium to high socioeconomic sectors of the population [[15]: 17].

The present paper has two broad aims. First, the paper seeks to highlight the extent to which the social location of migrants in the host society alters or restructures the cultural practices people bring with them. In the paper, the critical realist perspective represents a way to address 'the missing link in medical anthropology.' Second, taking *hanbang*^i ^medicine in the Korean-Australian community as an illustrative case, the paper explores the transformation of the dominant biomedicine in Australia as a result of the influx of ethnomedicine in the era of global capitalism and global movement. The terms ethnomedicine, complementary therapy, traditional medicine, alternative medicine and complementary and alternative medicine (CAM), carry different connotations for different people [16]. In this study the terms are used interchangeably.

### Theoretical considerations

Gray's [17] recent categorisation of four perspectives on 'unconventional therapies' is useful in the present context for highlighting the contrasting approaches to complementary medicine: the biomedical, the alternative, the progressive and the postmodernist. The primary focus of *the biomedical or dominant health perspective *in the West is on physiological disease processes. Curing disease through the scientific control of symptoms remains the primary task of dominant biomedicine. Proponents of biomedicine generally show hostility towards complementary therapies for a number of reasons. First and foremost, in contrast to biomedicine, which is preoccupied with scientific medical concerns, complementary therapies are not based on scientific rigour. As well, complementary therapies are practised largely in a deregulated market and thus have the potential to be misused or abused. Moreover, the key principles of health and illness that underpin complementary medicine are incompatible with the biomedical assumptions that have been developed through scientific rigour over time [[17]: 58]. At the same time, however, even among advocates of biomedicine, there are those who realize that complementary therapies will not disappear of their own accord, and that perhaps there may be a place for ethnomedicine in the wider context of health care.

The popularity of complementary therapies has compelled many within the biomedical community to reflect on their continued appeal, what they offer to patients and why there is an increasing dissatisfaction with biomedicine. Some advocates of dominant biomedicine encourage physicians and nurses to react sensitively to patients who express interest in complementary therapies. Notwithstanding these attempts to accommodate complementary medicine, biomedical proponents continue to argue that objective and non-judgemental evidence will orient patients to the validity of the biomedical and allied practices [[17]: 59].

Gray's *alternative perspective *values a diverse range of healing approaches. Proponents of natural medicine are in support of the view that disease processes are more than symptoms of ailing parts of the body, but are also symptoms of 'underlying, systemic issues' [[17]: 59, [18]]. The third perspective which Gray labels, *the progressive view*, supports both biomedical and alternative approaches. In contrast to the previous two approaches that focus on opposite extremes in the health care practice continuum, the main characteristic of the progressive view is that it endeavours to present 'a general even-handedness' that allows for all therapeutic possibilities, and, more specifically, to extend 'the application of research methods and even research funding, to the alternative domain' [[17]: 63].

Finally, the *postmodernist view *challenges the key assumptions of the dominant modernist cultures, including the notion of an autonomous human subject/agent, the idea of accumulative progress, as well as the idea of universally valid objective knowledge. Postmodern proponents of complementary therapies are concerned with the 'particular, as opposed to universal, truths'. They are interested in 'encouraging the articulation of perspectives on health care practices besides those of the dominant biomedical approach' [[17]: 70]. Although postmodernists encourage a high level of medical pluralism and generally are supportive of the benefits of complementary alternatives, the extent to which it is possible to achieve plurality is open to debate [8]. Gray's four approaches serve to highlight the deep division and scepticism that persists in attitudes to complementary medicine.

Contrary to the popular stereotypical impression of the two health care systems as being opposite and contradictory, the available evidence confirms that the relationship between dominant biomedicine and ethnomedicine is dynamic and inter-related, each influencing the other [1,19,20]. The distinguishing features of complementary/alternative medicine and dominant health care are caricatured in popular discourses in a manner that exaggerates the distinctions between biomedicine and alternative medicine. However, there is considerably more overlap between the two approaches than critics have been prepared to acknowledge.

The two systems of health care often operate in parallel rather than as opposites. The extent to which ethnomedicine receives prominence in particular social contexts cannot be explained simply by drawing attention to physiological treatment outcomes, but is an outcome of the very same political and social factors that have secured in other societies the dominance of biomedicine. These socio-political factors have to do with the diverse health care needs of individuals, the nature of the care that is performed and the professional controls adopted by the practitioners of each mode of care. In this paper we propose that a critical realist perspective may assist in broadening our understanding of why ethnomedicine has prospered in the West.

In the critical realist framework that we are advocating, individual agents facing pre-existing social climates undergo changes within the restraints of the social structure, and that social structure in return also transforms itself and reproduces itself in part. To the degree to which individuals either yield to social forces or resist them, societal structures are either reproduced or transformed. In the present context, we draw attention to Bhaskar's [10] transformational model of social activity to indicate that a similar process can be observed in complementary alternative medicine's reproduction and transformation as it is enabled or restrained by the given social and historical context. Any health care method or health remedy gets adopted, is transformed and consequently prospers, disappears or reappears in response to socio-political circumstances. The mix of dominant biomedicine and ethnomedicine in a particular society is not constant but varies, with some societies favouring biomedicine while others are more accepting of ethnomedical alternatives.

The carer-patient or doctor-patient relationship, for example, to a significant degree shapes both the practice and choices of health care. The carer maintains expertise or qualifications obtained through traineeship, while the patient ultimately responds to options made available by the carer. Medical service is a commodity that is sold and bought. All the medical services involve a period of traineeship, advertising, special tools to be used for treatment and drugs. The reason why people pursue medical training is not only because they hope to achieve personal aesthetic goals, but also because they see the possibility of finding a place of employment. This applies also to complementary therapies [21,22]. The supply of necessary tools and drugs implies the involvement of pharmacists, drug manufacturing companies and distribution.

Biomedical and other health services usually take place in a clinic; where a patient is unable to come to the clinic s/he is visited by the health worker. Providing the service in an established clinic is a consequence of the professionalisation and commodification of medicine. As long as the patient is in the clinic, surgery or hospital, carer-patient relationships tend to persist, irrespective of whether they have a preference for biomedical health care or ethnomedicine [8].

Health workers tend to medicalise the disease even when it is a socially constructed reality. In recent years some health services, especially those offering psychiatric treatment, have utilized social scientific knowledge and socio-economic factors concerning the client as a method of curing disease. However, the diagnosis and treatment by health workers are primarily based on physiological knowledge.

There is always competition to attract clients/patients not only within each medical tradition, but also between different medical services in order to enhance the reputation of each against competitors [23]. The latter kind increases the level of particularism of each institution. In other words, health care systems have as many things in common as they have differences: they compete for market superiority and social branding [24]. This competition is also closely tied to winning or maintaining social acceptance and consequently to receiving financial reward.

Despite the biomedical doctors' claim to authority, other health workers likewise attract a significant proportion of patients both in the East and West. The fact that biomedicine has gained greater proportion of a nation's medical system has to do with coercive and regulatory factors of various kinds rather than with natural development and demands from the population [[23]: 303].

Finally, a high level of professionalisation can be observed within both biomedical and complementary/alternative services [25]. Providers of health services try hard to maintain well-organized professional associations through which to keep control over who is licensed to practise. Associations maintain close links with higher education sectors (e.g., medical schools), health planning agencies, public health agencies, insurance companies and hospital administrators [[26]: 124, [27]].

Contrary to popular discourses that position dominant biomedicine and ethnomedicine at opposite ends of the pole and disallow the possibility of coexistence, the point that is argued in this section of the paper is that a similar set of processes may be observed controlling health care, the carer-patient relationship, the training of health practitioners and maintaining monopoly over expertise in both dominant biomedicine and ethnomedicine. The science that underpins dominant biomedicine and ethnomedicine may be different; however, the social characteristics of the two have more similarities than differences. The level of prestige accorded to each very much reflects time and place; people know to whom they turn for health care and why. South Korean (Korean hereafter) immigrants living in Australia certainly recognize the different *means *that biomedicine and ethonomedicine use to deliver health care, but in their view, the *ends*, that is, the health outcomes for individuals of the two systems of health care ultimately are compatible.

## Methods: sampling, data collection and validation and data analysis

This article draws on research data collected between 1995 and 1997 by Han for an earlier study of the use of biomedical and traditional medicine by Korean-Australians in Sydney [see[19]]. A total of 120 interviews were conducted with a range of participants, including 8 biomedical doctors, 1 pharmacist, 2 physiotherapists, 8 traditional health professionals (herbalists and acupuncturists), health food shop owners, Korean community leaders and Korean migrant men representing a range of socio-economic backgrounds and migration patterns – 17 amnesty; 14 skilled and 9 business migrants.

In most studies of traditional medicine amongst immigrants, the socio-economic context of traditional health care and the practitioners' views are not incorporated with the exception of Pang's [28] study. Han [19] attempted to cover as many providers of *hanbang *medicine as possible. A convenience sample was taken of practitioners and health service users introduced to the author through local networks until data saturation was achieved [cf. [29]]. However, it was made sure that the sample was not all from the suburb of Campsie, where a large number of Korean owned businesses are located.

The author (Han) had already done some empirical work on a related project in the Sydney Korean community [i.e.,[30]] and had a considerable knowledge about this study's aims and appropriate methods. The author, who did not live in the Sydney area, aimed throughout the study to take a 'native-as-stranger' approach [31]. Informal interviews were often possible as a result of being invited to stay on to take refreshments with interviewees after their formal interview. In general, the author considers that interviewees regarded the interview as an opportunity to share their views of life in Australia with a Korean researcher.

A consent form, either in English or in Korean, was given and the interviewee was asked to sign it. Interviews took place in a place of the respondents' choice, this being mostly their homes, and were conducted in Korean. Semi-structured interview schedules were prepared around the reasons for emigration, the process of adjustment, work involvement, maintenance of health and health care use. Starting with broad questions, the researcher asked the interviewees to elaborate some of their answers in more detail. The researcher restrained as much as possible from talking as a way of minimising his influence on data quality. Most interviews lasted about one to three hours. The total number of interviewees for the broader study reached over 120. Seventy formally arranged interviews were tape-recorded and then transcribed into a full text report for analysis.

A previous study of the Korean community by the author [30] helped inform many aspects of this study. The comparison of the perspectives provided by the users [see [1]] and providers of health care [20] has been an important source of cross-checking the data so that the validity has been increased [cf.[31]: 210]. The cross-checking has also served as a stimulus for examining, for example, discrepancies between the views from the users [see[19]] and providers are observed rather than merely being regarded as 'distorted' or inaccurate [32]. The findings from the interviews with the providers of *hanbang*, presented in this paper, are remarkably in common with those of the users. Qualitative methods were used to analyse interview texts. Most importantly, a grounded theory approach [[33]: 111] was used to identify, develop and test themes arising from interviewees' accounts.

The following methodological issues have also been considered in designing the study: what constitutes the characteristics of the relations between complementary health practitioners and their patients in the Korean community?; what properties must exist for the use and provision of complementary therapy among Korean immigrants, both at the levels of the Korean community and broader Australian community? These are specifically critical realist inquiries to seek influential or relevant qualities beyond what is immediately given or observable [34,35]. From a critical realist perspective, social reality has ontological depth and consists of domains of actual, empirical and real. That is, *actual *phenomena of the use and provision of complementary therapy in the Korean community may have produced perceived (*empirical*) views which can be further explained by relevant social structural or *real *underpinning factors.

## Discussion

### Traditional medicine and economic development in South Korea

Economic development is often positively related to the expansion of biomedicine. However, the expansion of traditional or non-Western medicine has not always accompanied economic prosperity. Moreover, official recognition of traditional medicine together with biomedicine is rare with the exception of China and India. However, Korean society, which has experienced rapid industrialisation and development over the last half a century, has observed the revival of *hanbang *or traditional medicine as well as its official recognition by the government, nearly as much as in China [26,36,37].

Biomedicine gained its particular significance during the Japanese colonial period (1910–45) and this trend continued following the end of the Japanese regime and the start of the American military government in South Korea (1945–48). Under the limited resources provided by the American aid program, economically better-off Koreans were encouraged to seek services from the private health sector and this accelerated the commodification of medicine to a significant degree [37].

Up until the 1970s when biomedicine had secured greater legitimacy in Korean society, both the preventive and curative natures of *hanbang *medicine had been maintained, although *hanbang *medicine had not experienced any significant expansion or decline. Eventually the powerful efficacy of cure-oriented biomedicine did threaten the existence of *hanbang *herbal medicine. As the Korean economy became more influential in the international market and a large proportion of Koreans started to enjoy the affluence of the 1980s, Korean *hanbang *medicine quickly adapted to accommodate affluent middle class values. *Hanbang *herbal medicine was prescribed and sold by *hanbang *doctors for many reasons. However, over the last few decades, *hanbang *herb medicine has been thought of primarily as something to restore the weakened 'whole body' rather than a particular part of it. *Hanbang *herb medicine has become almost equivalent to tonic or restorative medicine (*poyak*). The demand for *hanbang *tonic medicine in Korea has been extraordinary among wage earners as well as the well-to-do. It has become known as a natural and side-effect-free method to restore or sustain health, i.e., the fundamental capacity to work [38,39]. In fact, *hanbang *professionals had a relatively low level of education and *hanbang *had no option but to sell *poyak *since it could not compete with biomedicine in curing disease. In this process, *hanbang *medicine had already started losing its market share of curative medicine even before the start of the intensive industrialisation of Korea in the 60s and 70s.

The Korean economy started to grow in the early 1970s, however, there was a shortage of biomedical personnel and the limited access to biomedicine became apparent. The upper and middle classes turned to biomedicine for the purpose of curing their ills, while using *hanbang *for the purpose of preventing illness or maintaining health. Those from the lower class continued to use informal care such as acupuncture, herbal and folk remedies, mostly for the purpose of curing. The latter had only limited access to biomedicine due to its high cost, although access has improved in recent years, that is, many years after the introduction of the government's health scheme for all [40].

As people's health in industrialising Korea has become the means for something else rather than an end in itself, it has become more essential to sustain acceptable health than was the case in agricultural Korea. Having good health is a basic requirement for every member of the work force in Korean capitalist development. In this context, the nature of *hanbang *herbal medicine which was considered to be both curative and preventative in the past, has changed its character, becoming primarily 'preventive.' This conforms to the needs of the middle and upper classes. *Hanbang *herbal medicine is now considered primarily restorative or tonic medicine for the mainly better off people who can afford it. The financial affordability of consuming *poyak *regularly has provided people with a sense of pride. This transformation of herbal medicine is part of a new strategy to overcome the dominance of scientific medicine in the context of rapid Korean industrial development. However, along with the seemingly affluent Korean economy since the 1980s, working class Koreans also have come to use *hanbang *herbal medicine frequently. When they could not afford it, they felt a sense of deprivation. The increase in demand for, or revival in use of, *hanbang *tonic medicine has been extraordinary over the last two decades. Several social factors may account for this revival of interest in *hanbang *in Korea [40].

The dramatic population drift from rural to urban areas started under the government's development program to increase cheap and disciplined labour in industrial centres in the 1960s had many unanticipated social consequences. The move was accelerated by the devastation of the rural economy resulting from the government's policy of keeping the price of rice low. The policy was meant to maintain the low living cost of factory workers so that low-priced Korean products could be competitive in the international market. The concentration of medical and educational facilities in urban areas also was instrumental in stimulating rural migration. This process resulted in the explosive increase in the proportion of wage earners. It also generated growth in demand for biomedicine for the purpose of curing disease, as well as the use of *hanbang *herbal tonic medicine for the purpose of sustaining health.

Over the last few decades, the demand patterns of both biomedicine and herbal medicine in Korea have influenced the health care choices of Koreans who have migrated. The increase in demand for herbal medicine was facilitated by the availability of information through the internet. *Hanbang *medicine as a tonic is now used by overseas Koreans engaged in physically demanding work and by those who suffer long working hours, for the purpose of boosting their health [1].

The increase in the number of *hanbang *medical courses at universities was spectacular: from two in 1972 to eleven in 1997 [[1,41]: 51]. Such courses have been among the most competitive courses to enter. Many first year students of *hanbang *medical courses have included postgraduates with Master's or doctoral qualifications in other disciplines. The conflicts between *hanbang *doctors and pharmacists over who should have the right to prescribe and disseminate *hanbang *herbal medicine, which has become an expensive commodity, involving the students of both professions at universities, have been serious in the health sector in Korea in the last two decades [36]. It is worth noting that *hanbang *has become highly institutionalised and reengaged in curative medicine. For example, some leading *hanbang *practitioners organised experimental treatment of severe diseases by applying both *hanbang *and biomedicine and requested that biomedical practitioners participate in their experiments and practices of cooperation. It is also interesting to note that the phenomenon of restorative medicine has not been exploited by *hanbang *only. For example, *Bakasu*, a popular tonic water drink in Korea since 1963, has marked one of the highest sales in Korean pharmaceutical market. BCom, a vitamin product, has also been a best selling item since the 1960s.

What is also at operation may be the process of the integration of both biomedicine and hanbang as well as the transformation of hanbang into the biomedical system. *Hanbang *has changed towards a type of medicine producing more profit. More profit could be made by changing herbal medicine as a whole into a profitable form, which has been supported by increasingly institutionalised medicine.

Under the pervasive capitalist mode of production, the health of wage earners has not necessarily improved and possibly declined. *Hanbang *and biomedicine concentrate on the individual body and neither *hanbang *nor biomedicine actually considers social origins and constraints on health and well-being. Both rely on medication, not on social, political and economic change to bring about improvements in health and well-being. Both heavily individualise health problems [19].

### Immigrant life and the health of Korean-Australians

The major flow of Korean migration to Australia started in the early 1970s. Several hundred Koreans, mostly from working class backgrounds who had worked as contract labourers in South Asia, the Middle East, or former West Germany prior to migrating, settled in Australia as 'amnesty migrants' and were subsequently followed by their families from Korea. While the migration of Koreans from middle class backgrounds continued in the 1980s and 1990s, a large number of Korean migrants in the 1980s were admitted under the category of skilled migration. A significant number of Koreans who arrived in the 1990s were business migrants. These inflows of Korean migrants from different backgrounds reflect developmental stages of Korea as well as those of Australia, allowing individuals to utilise given social and economic circumstances to meet their needs [42].

Like other migrant groups [43], Korean migrants who came to Australia settled in close network clusters with common patterns of adjusting to their new homeland. Amnesty migrants are working hard, generally holding two or three jobs at a time. They arrived in Australia from many countries on short-term visas and most found employment in Australian factories almost immediately upon arrival. However, their modes of employment and difficulties of settling in a new social environment severely undermined their quality of life. Their health rapidly deteriorated, with many Korean migrants reporting that friends and family members were dying even around the age of sixty or younger. Despite the general ill health of the amnesty migrants, on the whole they remain satisfied with their new life in Australia. This is largely because they have suffered the least from status anxiety (resulting from the discrepancy in social status prior to and after their immigration to Australia) in comparison with other Korean migrants. However, the 'mental trauma,' to use the words of one Korean doctor in Sydney, cannot be underestimated. The psychological difficulties and stresses they went through as wandering immigrants in many parts of the world, and leaving their families in Korea, have been enormous [cf. [44,45]].

Few regret migrating and many are of the view that, notwithstanding the difficulties that they have experienced, they would have fared much worse had they stayed in Korea. In Australia, they are rewarded commensurate with the labour they contribute. Their children receive tertiary education in Australia. The combination of hard work and the favourable economic environment in Australia has enabled many to accumulate sufficient capital to start small businesses, such as cleaning contracts or take-away food shops. Relative to other ethnic groups reported in the 1981 Australian census, home ownership is higher among Korean migrants as is their average household income. However, these social successes have been achieved at the cost of their health; hard work and physically demanding labour have taken their toll [1].

Manderson [[46]: xiv] asserts,

"when compared with the general population, non-English-speaking migrants [in Australia] have the lowest income, the highest incidence of poverty, and the highest rate of unemployment, other than Australian Aborigines, and are the most at risk of industrial accidents and related occupational health problems."

Furthermore, like other migrant groups, unfamiliarity with the health care system and reticence to approach health care professionals prevents many from using the health services and their health suffers. Like other migrant communities, Korean newcomers do not consult health professionals as often as they should, even at times of sickness. Amnesty immigrants who had entered Australia without either a work visa or permanent residency, were not entitled to state-provided Medicare benefits, and feared contacting health officials. Even after they became entitled to Medicare, they did not utilise the service as much as they could have done. There were no Korean speaking biomedical doctors until 1987 [[47]: 350]. This had virtually prevented them from using the service or minimised their use of the service. Skilled migrants, by contrast, who entered Australia as legitimate immigrants were better educated and brought some capital. Most skilled migrants were relatively successful in Korea, but chose to migrate because they were not satisfied with their circumstances in their homeland, including the pollution in the cities, the widespread corruption, and the poor quality of life. They left Korea in search of better opportunities for themselves and their children. Skilled and business migrants who settled in Australia found it difficult and stressful to bear the transition of settling in a new environment, gaining employment, and adjusting to a different cultural context. This status anxiety was a matter of great concern for them, as they were used to the highly hierarchically-oriented structure of Korean society where they had professional positions or potential [48].

The sluggish economy in Australia at the time of their arrival in, and since, the 1990s, exacerbated their deprivation and made it even harder for them to accumulate the necessary capital to start small businesses. Professional expertise obtained prior to their immigration did not help skilled migrants and their poor command of English even further reduced opportunities for employment. Economic efficiency and high productivity are essential in the employment market. The majority of skilled migrants remained as wage earners, doing manual work such as cleaning or becoming factory hands. A small number who managed to utilise their professional expertise saw little prospect of promotion. Although skilled migrants brought a sum of capital, they did not seem financially far better off than the amnesty migrants. Despite the popular and politically promoted multiculturalism, structural racism has been deeply embedded in Australian society; in this respect skilled migrants were only marginally better off than amnesty migrants [49].

Skilled migrants, who engaged in doing menial work or running small businesses, experienced 'loss of face' and low self-esteem. Thus, the health of skilled migrants declined both physically and psychologically. The so-called *iminppyong *(immigrant disease, commonly known in the Korean community) was as pervasive among skilled migrants as it was among amnesty migrants. Like amnesty migrants, some skilled migrants died unexpectedly and prematurely of over-work [1].

Lack of English language proficiency and limited understanding of Australian business culture discouraged skilled migrants from pursuing business activities and frustrated their business efforts. They often witness that Australia is not a place in which to make a profit, but rather a place in which to spend what they had when they came. Only about a third of business migrants became engaged in businesses, ranging from takeaway food shops to Korean food-importing companies. Some went bankrupt. There are also some who left a fair proportion of their capital in Korea when coming to Australia. In these cases, while the wife and children live in Australia, the man would spend a significant proportion of the year in Korea to manage his business (*Appendix, iv*). Many came at the age of retirement or close to it, wishing to have a comfortable retired life. Some still have a business in Korea managed by others, which is the major source of their income. Others live on the interest of their bank balance and those who came with limited capital have supplementary income from government-paid welfare allowances [51].

This group appeared to have relatively better health than any other group of people in the Korean community prior to their emigration. They had more resources with which to meet their health needs. Like skilled migrants, the major factors which have adversely affected their health, prior to coming to Australia, were long working hours, heavy drinking, smoking and irregular meal times. Business migrants mentioned that they generally improved their health after coming to Australia. After all, they are not heavily involved in work. Some of the frequent physical health problems include, 'tennis elbow' or back pain, caused by playing golf. Business migrants spend lots of time at the golf links. This has probably enhanced their physical health. However, golfing everyday may be pleasurable for the first few months; but problematic if it continues for a longer period. Not being able to carry out what they had wished to do is a major source of psychological distress. The analysis of the subjective views of three different groups of Korean men on their health status could be described as in Table 1 [[1]: 111].

**Table 1:** Physical and mental health of Korean men in Sydney

	Amnesty migrants	Skilled migrants	Business migrants
Physical health	-	-	+
Mental health	+/(-)	-	-

- relatively bad; + relatively good; +/(-) mostly good, but some bad

As aforementioned, no Korean speaking biomedical doctor was available until 1987. Consulting a biomedical doctor was out of the question for most amnesty migrants because of their fear of being found to be illegal migrants. When they became permanent residents they began to consult doctors from Indian, Chinese or Egyptian backgrounds. The use of biomedical services gradually became common and many Koreans now seek regular health check-ups once or twice a year. The frequent use of medical services has been made possible under a state-subsidised health service. Thus, the use of biomedicine has been intensified by amnesty migrants who had benefited little from Korean medical facilities available prior to their emigration. However, there are also some Koreans who find it hard to make time to pay a visit to the doctor. They seek medical advice only when they cannot cope with their ill health. In some cases, their lives have ended soon after hospitalisation.

### Korean traditional medicine in Australia

As of January 2005, there were more than a dozen *hanbang *doctors in Sydney practising herbal medicine or acupuncture in the Korean community [[52]: Jan. 14]. Complementary alternative medicine is not only an alternative in substance, it is also alternative in its approach to patients. It makes greater provision of time for consultation and involves more listening to the patient [cf. [53]]. Complementary alternative medical practitioners hear and ask about the clients' melancholy, inquire about routine aspects of their lives, their joys and frustrations prior to coming to Australia and following their arrival. They show interest in understanding the plight of women migrants and their double burden of migrant life, their imperative to hold two or more jobs to keep their families fed and clothed, and the impacts upon their health. These are some of the reasons for going to *hanbang *doctors as claimed by the interviewees in the study.

Employing the concept of global 'millennial' or 'casino' capitalism [54], Adams [55] illustrates the ways in which market interests characterise or influence scientific characteristics of medicine with reference to medical 'truth.' This is in part how *hanbang *herbal medicine has constantly reconstituted its characteristics in the context of casino capitalism.

There was no herbal doctor or acupuncturist in the Korean community in Sydney until 1980. Although the small number of legitimate migrants mostly used biomedical services, illegal migrants tended to use herbal medicine and acupuncture in Chinatown only when they were desperate with acute pain. Until the end of the 1970s, the Korean community in Sydney was too small to attract a herbal doctor or acupuncturist from Korea. When the first *hanbang *clinic was opened in 1980, the amnesty migrants began to use acupuncture therapy to overcome quickly their problems which were predominantly related to their involvement in manual work and long work hours. They have utilised *hanbang *tonic medicine as a way of boosting their health so that they could pay back their mortgages on their homes in a minimum period. They have also used health food to sustain their health. The use of health food among the amnesty migrants seems to be an extension of their use of various pharmaceutical products in the form of 'nutritional tablets' which were popular in Korea in the 1960s and 1970s when malnutrition was a common problem for most Koreans [19,20].

The 'freely' available state-provided medical service in Australia encouraged the skilled migrants to visit biomedical doctors. Some skilled migrants could not be bothered to take *hanbang *tonic medicine, however, according to *hanbang *practitioners, a significant proportion of others take it regularly [20]. The reason for its consumption is again frequently related to their involvement in manual work and long work hours. When the major flow of business migrants came in the 1990s, a variety of medical services were already available in the Korean community. Although they might have had easy access to biomedicine in Korea, the use of it in Australia intensified, thanks to the state-provided Medicare. The free time they 'enjoy' seems to encourage business migrants to visit biomedical doctors as often as necessary [19].

The practice of *hanbang *herbal medicine and acupuncture has become increasingly popular since the arrival of skill-based and business migrants, which included university-trained *hanbang *doctors, self-taught doctors or those trained under traineeship and herb dealers. The demand for herbalist and acupuncturist services increased because of the non-existence of Korean speaking biomedical doctors and migrants' ill health caused by their involvement in the heavy manual jobs at the bottom of the Australian labour market. The use of tonic medicine was especially thought to boost their health while they were engaged in manual work such as welding and cleaning. Herbal medicine or *poyak *has frequently been used by both amnesty and skilled migrants [19].

Most *hanbang *doctors had middle class or professional jobs in Korea, but wanted to live a more comfortable life in a better social and natural environment. Thus they have come as business migrants since the late 1980s. The business plans they submitted to the Australian Embassy were not about herbal medical practice. However, planning to practise medicine in the Korean community, most of them brought herb remedy cabinets at the time of entry. As immigrants, *hanbang *doctors and acupuncturists have generally experienced the same difficulties as other Koreans. However, a unique aspect of this group of business migrants is that they came with *hanbang *medical skills which are readily marketable to fellow Koreans [20].

There are also other factors which Korean immigrants have faced. Firstly, there is the problem of the gap between expectations and reality in Australia. Upon their arrival, Koreans were so happy to have left behind the highly competitive life in Korea and to have come to the land of 'opportunity.' However, they soon realised that there was little chance to have their dreams come true in a short span of time. It does not take long for this realisation to reap its toll on self-esteem. Secondly, there is a gap between Korean and Australian norms and cultures. Korean ones are linked to their cultural background and rapid social change based on industrialisation and urbanisation, whereas Australia has already seen advanced capitalism. Many Koreans in Australia were born in rural areas and experienced peasant lives. As they have brought their own or Korean cultural attitudes, thinking patterns and values, they have some difficulties adjusting to the dominant culture of Australia [42].

The implications of continuing involvement in physically demanding work and the experience of psychological distress have been expressed by both the users and providers of medicines. It is understandable that victims of ill health look to utilise all available health services. The first group of health practitioners they would consult is the state-subsidised biomedical doctors. If their health problems could not be resolved, biomedical doctors often refer patients to other health practitioners such as acupuncturists or Korean herbal doctors. In other cases, Korean patients with scientifically unidentifiable fatigue or work-related tiredness, would go directly to *hanbang *doctors. It seems natural that Korean immigrants in Australia who are familiar with Korean traditional herbal medicine are inclined to it when the limits of biomedicine are realised [19].

Most biomedical doctors in the Korean community are the offspring of amnesty migrants whose lives have been affected by the Korean, international and Australian capitalist development processes. The amnesty migrants have commonly mentioned that they almost sacrificed their lives in order to make it possible for their children to climb up the social hierarchy. In addition to this, tertiary education benefited a small proportion of Korean students. Medical practitioners have become one of the more privileged groups in the Korean community. Korean speaking doctors made a significant contribution to the health needs of Koreans in Sydney. It is an undeniable fact that doctoring in the Korean community is lucrative. Korean *hanbang *doctors seem to have pursued 'doctoring as a business' in a fairly proactive manner [cf. [56]]. The services provided by biomedical doctors in particular were accorded legitimacy and rarely questioned.

*Hanbang *continues to appeal to Korean-Australians as it does to wage earners in Korea. *Hanbang *doctors who immigrated brought to Australia their craft and skills in using traditional medicines. Their expertise in using traditional medicines meant that they were immediately differentiated from other Korean migrants. Despite claims of *hanbang *doctors that the service they provided was more natural, free of side effects and appropriate for human health in comparison with that of biomedical doctors, their service was astonishingly similar to that of biomedical doctors. *Hanbang *doctors were quick to attribute health inequalities to poor food habits and individual behaviour. Like the biomedical doctors, *hanbang *doctors were prone to medicalise the patient's illness and often did not incorporate knowledge of the wider social factors that impact on health and illness. It was not surprising that they showed little interest in the social construction of health, illness and medicine. Some *hanbang *doctors admitted a preoccupation with the pursuit of 'doctoring as a business'. Indeed, a number of *hanbang *doctors had established successful and profitable businesses servicing the health needs of Koreans living in Sydney [20].

### Biomedicine and ethnomedicine in Australia

Coulter and Willis [[16]: 588] note that ethnomedicine 'has become a widely used form of health care', with up to 42 per cent of Australians reporting using traditional medicines. MacLennan et al. [57] estimate that complementary therapy/medicine has developed into a thriving service industry in Australia, generating in excess of $2.3 billion annually. The increasing popularity of complementary medicine and a growing realisation of the limitations of biomedicine have led many physicians to adopt a more favourable attitude towards complementary medicine. Some family physicians frequently refer patients to unconventional therapies [58,59]. The extent to which a biomedical physician refers patients to complementary therapists and how a physician perceives complementary therapies can cause unwanted or subtle animosity between physicians [60]. Referral to complementary therapists can happen especially when biomedicine does not have any effective prescription to offer, for example, for chronic fatigue syndrome. Indeed, complementary medicine thrives and grows at the limits of dominant biomedicine.

A 1992 survey of health care providers highlighted that there were 2,000 Australian general practitioners using some form of complementary therapy in their medical practice [Sali cited in [61]]. The number increased to about 4,000 in the mid-1990s. In 1997, about 20 per cent of Australian general practitioners incorporated at least one kind of complementary medicine in their treatment of patients, and about 50 per cent expressed an interest in acquiring additional training in using complementary alternative medicines [62]. At the professional organisational level, the Australian Medical Association has issued a position paper stating that evidence-based complementary and alternative medicine has a valuable contribution to make towards mainstream medical practice and that medical practitioners should be informed well enough to be able to provide patients with appropriate advice [63,64]. The joint working party of the Royal Australian College of General Practitioners and the Australian Integrative Medicine Association endorsed the AMA Position Statement and argued for continuing professional training opportunities for 'safe and ethical practice' on 'evidence-based complementary medicine' [65]. As Pinn [66] previews, the attempt to integrate complementary alternative medicines into broader clinical practice will continue. As Cohen [[64]: 646] suggested, there are barriers for greater integration between the two camps: complementary/alternative medicine 'practitioners may feel threatened by [biomedical] doctors invading their turf, and doctors may be hesitant to collaborate with CAM practitioners.' A high degree of integration may be achieved especially under the Australian government's recently released MedicarePlus package in 2004, supporting allied health and complementary alternative medical services, through a rebate for service, when providing complementary care in collaboration with general practitioners.

However, as with Tibetan medicine in urban Mexico, a selective regularising, criminalising, and commodifying of complementary/alternative medicines is evidently at work in Korea, Australia and elsewhere and the competition for legitimacy in the arena of health care is likely to continue [cf. [2], cf. [67]]. The move to integrate the two health care systems remains problematic. The biomedical profession's open approach to complementary/alternative medicines in recent years may be considered as being a reluctant easing of market monopoly in response to the public's demand for traditional medicines. This trend is too significant to ignore [57,68,69]. It is particularly interesting to note that the phrase 'unorthodox medicine' is now commonly used to refer to 'alternative medicines' used in the past, but the negative labelling of traditional medicines is no longer justifiable even among the biomedical professionals [[70]: 275]. Although there is clear benefit of integrating complementary/alternative medicines into general clinical practice, reducing the barriers and appropriate credentialing of traditional practitioners will contribute towards an effective and better relieving of human suffering [64,71,72].

The examination of the use of traditional medicines among Korean-Australians presents us with a useful micro focus for understanding the links between dominant biomedicine and complementary alternative medicines. While the distribution of traditional health practices, on the whole, remains marginal to the medical outlooks and practices of the majority of biomedical doctors in Australia, traditional medicine occupies a prominent place within the migrant community. The study has highlighted that for many in the Korean-Australian community dominant medicine is the default option for health care, and it also highlights an absence of ambivalence either in seeking assistance from traditional medical doctors when biomedicine fails, or in supplementing their biomedical care with traditional care. At the level of the professions, and notwithstanding the significant extent to which some biomedical doctors have appropriated traditional medicines in their clinical practices and others who are prepared to recommend patients to traditional doctors, the two approaches to health care continue to be caricatured as representing conflicting ideologies of care. In the migrant community, however, we are presented with an ideal-type of how the two systems of health care can be integrated in ways that reinforce one another.

## Conclusion

Certainly the increasing popularity of complementary medicine in recent years is partly attributable to people's dissatisfaction with biomedicine [73] and thus poses a very real challenge to it [74]. Complementary medicine thrives and expands in the gaps of conventional medicine. Research suggests that users of alternative medicines are both aware of biomedicine's inability to cure many illnesses and are not always satisfied with biomedical treatment [75]. They are reacting to biomedicine's insensitivity to the needs of individual patients and its inability to respond appropriately to the social and experiential aspects of illness [76,77]. By contrast, complementary therapists are presented as being friendlier and more accessible than their biomedical counterparts. Their holistic approach endeavours to integrate the patient's and family's experience of illness to facilitate healing. Their method of cure may not be based on scientific rigour, but proponents note that it does contain 'demonstration effects'. A majority of complementary therapists in most non-Western countries maintain all the trappings and symbols of power they associate with western biomedicine, including 'stethoscopes', 'motorbikes' and 'wristwatches' [78]. In India, China and Korea, the non-orthodox medical systems are backed by formally recognised colleges, hospitals, research institutes, pharmacies and clinics. Other countries have a lower degree of professionalisation in alternative medicine, but in many Asian, African and Latin American countries, professional associations of traditional practitioners exist; training and practice take place in clinics; and commercial companies take care of the manufacturing and advertising of indigenous medicines [[79]: 194]. The popular use of alternative medicine in part reflects the increasing awareness of the limits of biomedicine or frustration with scientific approaches to health and illness. At the same time, however, complementary therapists have been proactive and have played their part in promoting their services.

In this paper we have argued that in seeking to explain the popularity and supply of alternative health care, it is important to go beyond the culture of each kind of health care itself and to take into consideration the changes occurring at societal, national and global levels as well as consequential individual response to the changes. New social conditions influence the choice of health care methods, including herbal/alternative medicine, health foods and what are often called New Age therapies. The transformations in the labour market and the global effects of the restructuring of work have led to increases in job insecurity, work-related stress and pressure on household budgets. These have also contributed to broader cultural changes, transformations in subjectivity and a pervasive attitude of needing to 'look after oneself' [cf. [27]: 331]. Further, transnational mobility of the general population and health professionals, the global supply of herbal or health food remedies, and readily available information through the internet have clearly accelerated the process in which alternative medicine has been commodified and become a significant player of global casino capitalism.

One of the serious deficiencies of non-political-economic studies or interpretivist perspectives in the disciplines of medical anthropology/sociology is the decontextualisation of the problems or subjects under investigation. Despite constant criticism of these approaches, decontextualisation has continued especially in the prosperity of, or polarity between, positivism, postmodernism, poststructuralism in the last two decades of the 20^th ^century. Deficiency has also been prevalent with studies which adopted 'macro' or crude 'structuralist' perspectives including some brands of Marxism. While the political economy perspective has clarified the relevance of political economic processes, their implications for the individual experience of illness and health are yet to be fully explored [[80]: 221].

The present paper has sought to demonstrate the explanatory power of a critical realist perspective in explaining the pervasive use of ethnomedicine by examining the case of the popular use of complementary and alternative medicine in the Korean community in Australia. We have illustrated the significance of influential factors in a given social and economic environment nationally and internationally, and individuals' or agents' work involvement to understand their physical and mental health, which are directly linked to their use of all available medicines. We have also discussed how the provision of medicines has been commodified and how the use and provision of ethnomedicine are related to the political economic aspects of a society. The paper illustrates the limitations of trying to explain the social origins of Korean settlement, work involvement, well-being and ill health, and the use and provision of biomedicine and herbal medicine without employing the insights provided by relevant political economic factors or critical medical anthropology/sociology.

The paper demonstrates that the combination of ethnographic study and political economy can assist researchers in avoiding the limitations of cultural explanations or adopting an individualistic approach in endeavours to understand the role of both agency and structure in social phenomena. This suggestion is old, but the employment of appropriate methodologies and the synthesis of interpretivist and political economic perspectives would enable researchers to avoid decontextualising the study participants. This will result in a fruitful outcome of academic research in the disciplines of medical anthropology and sociology as is evident in the past research e.g., [9,20,81-84]. How critical realism will equip itself with appropriate research methods and how it will translate into emancipatory and transformative practices will be revealed in time [85].

## Authors' contributions

GSH carried out the original research on the use and provision of biomedicine and ethnomedicine in the Korean community in Sydney. On the basis of his published papers, GSH prepared a draft. GSH and HB modified the draft in consultation with each other for original submission, also further developed the paper in similar methods in the light of the three anonymous reviewers' comments.

## Appendix

^i ^*Hanbang *literally meant Chinese method or more specifically the Han dynasty's medical method. Since its introduction to Korea in about 500 A.D., it has been indigenised to a significant degree. The Chinese characters of the word *hanbang *have been changed with the indigenisation of the medicine, and it now literally means Korean medicine. Thus, *hanbang *in Korea is quite different from Chinese medicine. A *Hanbang *personnel or a *hanbang *doctor, generally practising herbal medicine as well as acupuncture, is called a *hanuisa*, whereas a biomedical doctor is called *uisa*. That is, the term doctor is used for both *hanbang *and biomedical doctor, regardless of their formal training backgrounds.

^ii ^We are indebted to an anonymous reviewer for this information and insight.

^iii ^We have also taken the liberty of incorporating this insight from a reviewer.

^iv ^This type of living arrangement has gained a much more popularity in the last few years mainly for the sake of children's education. This does not necessarily involve the family's migration. About 10,000 school-age children left to study overseas in 2002, often accompanying their mothers [50].

^v ^A good number of Korean biomedical doctors in the community practise acupuncture.
